# Resource-Efficient Fusion over Fading and Non-Fading Reporting Channels for Cooperative Spectrum Sensing

**DOI:** 10.3390/s150101861

**Published:** 2015-01-16

**Authors:** Dayan Adionel Guimarães, Guilherme Pedro Aquino

**Affiliations:** National Institute of Telecommunications - Inatel, Av. Joao de Camargo, 510, Santa Rita do Sapucaí 37540-000, Brazil; E-Mail: dayan@inatel.br

**Keywords:** cognitive radio, cooperative spectrum sensing, decision fusion

## Abstract

Recently, a novel resource-efficient technique for the reporting channel transmissions in cooperative spectrum sensing was proposed. In this technique, secondary users are allowed to simultaneously send their local decisions to the fusion center, saving time and frequency resources. Expressions for the probabilities of detection and false alarm for the unitary-gain AWGN reporting channels were derived, while simulation results were given for both the AWGN and Rayleigh fading channels. Here, we provide an expression that is applicable to AWGN channels with different real-valued gains and to time-varying real-valued gains. A simple suboptimum receiver is proposed for the general complex-valued fading and non-fading channels, with an improved performance in the low signal-to-noise ratio condition. Numerical results are shown for both the AWGN and Rayleigh fading reporting channels, demonstrating the accuracy of the derived expressions and the attractive performance of the proposed receiver.

## 1. Introduction

In the cognitive radio (CR) [1] concept, unused spectrum bands in the primary (incumbent) network can be shared with secondary CR networks opportunistically. Spectrum sensing [2] is the task of detecting these unused bands, so that CRs can use them without causing harmful interference for the primary users. In order to increase the reliability of the decisions about the occupancy of a given channel, cooperative spectrum sensing has become the main alternative [2]. In cooperative spectrum sensing, individual CR or secondary user (SU) decisions or measurements are sent to a fusion center (FC), where the final decision upon the channel state is made and reported back to the SUs for subsequent channel use. A well-known decision fusion rule is the *K*-out-of-*M* rule, in which the FC decides upon the presence of a primary user (PU) when at least *K* among *M* secondary users declare an active primary user in the band of interest.

To send their local decisions to the FC, the SUs make use of a reporting channel, adopting some multiple access techniques, such as time division multiple access or frequency division multiple access. However, as the number of SUs grows, these multiple access techniques tend to require more time or frequency resources, reducing the overall spectral efficiency of the fusion task.

A number of attempts have been made for saving resources during the reporting of secondary user's decisions. For instance, in [3], the authors propose a report method to decrease the average number of sensing bits sent to the FC. In this method, only the users with high reliability are allowed to report their local binary decisions. A cooperative sensing without a dedicated control channel is proposed in [4]. In this proposal, the SUs send their local decisions to the FC in the same primary licensed user channels, which will potentially cause interference for the PUs. The authors of [4] suggest how to mitigate this interference in [5], where results show that it is possible to save the dedicated channel resources without sacrificing the sensing performance. In [6], a sequential-test is introduced at each SU for the local decision report. In this scheme, each SU reports its local decision only after having enough confidence. Therefore, each PU reports its decision at a different time, decreasing the necessary maximum bandwidth of the control channel. Numerical results show that the sequential test decreases the number of simultaneous reports and the probability of false alarm. In [7,8], a scheme, named sequential cooperative spectrum sensing (SCSS), is explored. In SCSS, the FC coordinates the reporting of local decisions in the SU network, choosing the SUs that will report their local sensing information. These SUs then randomly send their decisions until the condition required to make a global decision is satisfied at the FC. The number of sensing reports is reduced by adopting this technique. In [9], the authors use a ring-based distributed spectrum sensing model to reduce the required bandwidth in the control channel. In this scheme, each SU shares the local decision with its neighbor, sequentially and in a ring basis, and the last SU makes the final decision about the PU signal presence. In [10], the authors propose a final decision weighting scheme with a low average number of sensing bits sent in the control channel. This scheme uses an adaptive weighting scheme with double threshold energy detection based on the water-filling principle.

The energy efficiency is also an important aspect of the spectrum sensing and can benefit from most of the attempts for reducing the traffic in the reporting channel. For example, a cluster-based cooperative spectrum sensing is explored in [11–14], where the SUs are grouped into different clusters, and only the SU with the best SNR of the reporting channel is responsible for transmitting the cluster decision to the FC. Besides reducing the amount of data transmitted on the reporting channel, this technique also reduces the power consumption of the cognitive radio network. In [15], the energy efficiency of a soft-decision scheme is improved by mapping the quantization levels of the sensing information into the position of a single bit in different time slots of the frame structure. In [16], the final decision at the FC can be anticipated, preventing some SUs from sending their sensing information, thus reducing the average number of bits sent through the reporting channel and increasing the energy efficiency. A censored, truncated method with sequential spectrum sensing is proposed in [17], where an SU is forced to stop sensing when the accumulated energy of collected samples is in a certain region; then, its local decision is transmitted to the FC. Otherwise, if this SU does not detect a level of energy in the determined region, no bits will be sent to the FC. Therefore, this technique increases the energy efficiency in both sensing and local decision transmissions. In [18] is proposed a scheme in which only one SU will broadcast its local decision. The other SUs will object or agree with the first SU. The SUs that have a decision different from the announced one will send objection information to the FC, while the agreeing SUs will be silent during the reporting time. A significant amount of bits will not be sent to the FC, promoting the energy savings in the network.

The technique proposed in [19], which is now patented and no longer opened for public access, is the basis for this paper. In this technique, secondary users are allowed to send their local decisions simultaneously to the FC, improving the resource efficiency in the reporting channel. Expressions for computing the probabilities of detection and false alarm considering unitary-gain AWGN channels were derived in [19], while simulation results were given for both the AWGN and Rayleigh fading channels.

This paper capitalizes the idea reported in [19], giving extensions and providing a more detailed, general and deep system analysis, along with several new theoretical and simulation results. Furthermore, a new expression for computing the probabilities of detection and false alarm is derived, which is applicable to AWGN reporting channels with different real-valued gains and to time-varying real-valued gains. A simple suboptimum receiver is also proposed here for the general complex-valued fading and non-fading channels, with the potential for an improved performance in the low signal-to-noise ratio (SNR) condition. To the best of the author's knowledge, this is the first paper exploring the novel idea proposed in [19] with extensions.

The remainder of the paper is organized as follows: In Section 2, we present the system model and the main results from [19]. In Section 3, we derive an expression for computing the probabilities of detection and false alarm for fixed real-valued reporting channel gains. Section 4 provides numerical results and discussions considering fixed and time-varying reporting channels with real-valued gains. Section 5 is devoted to the receiver structure for the more general case of complex-valued reporting channel gains and to its performance improvement under the low signal-to-noise ratio regime. In Section 6, we give numerical results and discussions considering time-varying, complex-valued reporting channel gains. Finally, Section 7 concludes the paper and give some directions for future research on the present topic.

## 2. System Model and Main Results

As in [19], a cooperative spectrum sensing (CSS) system with *M* secondary users that simultaneously transmit their local hard decisions to the fusion center using binary phase-shift keying (BPSK) modulation is considered. Let *m_k_* represent the binary local decision generated by the *k*-th secondary user, with *m_k_* = 1 indicating the presence of a primary user signal (hypothesis *H*_1_) and *m_k_* = 0 indicating no active primary users (hypothesis *H*_0_). The baseband equivalent of the transmitted BPSK symbols with energy $\sqrt{E_{\text{b}}}$ is $s_{k}=(2m_{k}-1)\sqrt{E_{\text{b}}}$, and if *h_k_* is the gain of the reporting channel between the *k*-th secondary user and the FC, the received signal sample at the FC is given by:

(1) $$r={\text{∑}}_{k=1}^{M}h_{k}s_{k}+n$$

where *n* is the additive white Gaussian noise (AWGN) sample with zero mean, variance *σ*^2^ and power spectral density *N*_0_ = 2*σ*^2^.

It is not explicitly stated in [19] that the reporting channel gains are real valued or complex valued. In the case of real-valued gains, this means that the transmitted symbols are added coherently at the FC, which is intuitively satisfying under the assumption of pure AWGN channels or under fading channels with negligible phase rotations. However, these conditions can be met if the secondary users are sufficiently close to the FC, a situation that cannot be achieved in most of the practical cases. It is more reasonable to assume the general case in which the transmitted symbols may be added incoherently at the FC, *i.e.*, the reporting channel gains are complex-valued. Here, we give a unified system analysis, considering both real-valued and complex-valued channel gains.

It is assumed in [19] that the channel gains are known at the FC, which makes it possible that the FC arrives at the final decision upon the sensed channel state from *r*. Specifically, define a local decision vector **s** = [*s*_1_, *s*_2_, …, *s_M_*]^T^, and let *D*_0_ and *D*_1_ represent the sets of local decision vectors corresponding to *H*_0_ and *H*_1_, respectively. Applying the maximum likelihood (ML) decision rule, the fusion center will choose *H*_1_ if the following inequality holds:

(2) $$\underset{\text{s}\in D_{1}}{\text{∑}}\exp\left(-\frac{1}{2\sigma^{2}}{\left|r-\overset{M}{\underset{k=1}{\text{∑}}}h_{k}s_{k}\right|}^{2}\right)\geq \underset{\text{s}\in D_{0}}{\text{∑}}\exp\left(-\frac{1}{2\sigma^{2}}{\left|r-\overset{M}{\underset{k=1}{\text{∑}}}h_{k}s_{k}\right|}^{2}\right)$$

By assuming *h_k_* = 1, which corresponds to a pure AWGN reporting channel, the author of [19] has derived an expression for the probabilities of detection and false alarm at the FC. In such a case, the signal part ${\text{∑}}_{k=1}^{M}h_{k}s_{k}$ in Equation (1) follows a Binomial distribution with *M* + 1 values that can be represented geometrically as points in a one-dimensional space. The set of points $\left\{(2K-M)\sqrt{E_{\text{b}}},\ldots ,M\sqrt{E_{\text{b}}}\right\}$ corresponds to the event that at least *K* secondary users detect a primary user signal, and the set of points $\left\{-M\sqrt{E_{\text{b}}},\ldots ,(2K-M-2)\sqrt{E_{\text{b}}}\right\}$ is associated with the event that the number of secondary users detecting a primary user signal is less than *K*.

Define *P*_D,SU_ and *P*_FA,SU_, respectively, as the probability of detection and the probability of a false alarm at a secondary user terminal. Similarly, let *P*_D,FC_ and *P*_FA,FC_ be the probability of detection and the probability of a false alarm at the fusion center, respectively. From [19], the probabilities of detection and false alarm at the FC, after a simplification, are, respectively:

(3) $$P_{\text{D},\text{FC}}=\overset{M}{\underset{l=0}{\text{∑}}}Q\left[(2K-2l-1)\sqrt{\frac{2E_{\text{b}}}{N_{0}}}\right]\;\left(\begin{array}{c} M\\ l \end{array}\right)\;P_{\text{D},\text{SU}}^{l}\;{\left(1-P_{\text{D},\text{SU}}\right)}^{M-l}$$

(4) $$P_{\text{FA},\text{FC}}=\overset{M}{\underset{l=0}{\text{∑}}}Q\left[(2K-2l-1)\sqrt{\frac{2E_{\text{b}}}{N_{0}}}\right]\;\left(\begin{array}{c} M\\ l \end{array}\right)\;P_{\text{FA},\text{SU}}^{l}\;{\left(1-P_{\text{FA},\text{SU}}\right)}^{M-l}$$

where *Q*(*x*) is the Gaussian *Q*-function [20] defined by:

(5) $$Q(x)=\frac{1}{\sqrt{2\pi }}\int_{x}^{\infty }e^{-u^{2}/2}du$$

## 3. Real-Valued Reporting Channel Gains

Considering reporting channels with real-valued, but not necessarily equal gains, in this section, we first provide an algorithm for numerically computing the exact distribution of the symbols' amplitudes. Then, we describe how the decision thresholds can be computed according to a simplified version of the ML criterion. Finally, we give the expression for computing the instantaneous probabilities of detection and false alarm and describe how this expression can be applied for computing the corresponding average probabilities over a fading reporting channel with real-valued gains.

### 3.1. Probability Mass Function of the Received Symbols

If the reporting channel gains are different from one, the noiseless received symbols, *i.e.*, the sum ${\text{∑}}_{k=1}^{M}h_{k}s_{k}$ in Equation (1), can take one of 2*^M^* possible values and can be seen as the weighted sum of independent and identically distributed (i.i.d.) Bernoulli random variables. The exact probability mass function (PMF) of ${\text{∑}}_{k=1}^{M}h_{k}s_{k}$ can be numerically computed using the following reasoning. We have a discrete probability distribution over a set of 2*^M^* discrete numbers given by all possible combinations of the weighted SUs' decisions. The probability of each number is given by the probability of the vector of Bernoulli variables associated with the number, *i.e.*, it is the probability in the binomial distribution associated with that vector divided by the number of times that a vector with the given number of nonzero elements appears. This procedure is translated into Algorithm 1, where the matrix **S** with elements *S_k,l_* ∈ {0, 1}, *k* = 1, …, *M* and *l* = 1, …, 2*^M^* contains as columns all possible secondary user's decisions, and **b** is a vector whose elements are:

(6) $$\begin{array}{ll} b_{i}=\left(\begin{array}{c} M\\ i \end{array}\right)\;p^{i}{\left(1-p\right)}^{M-i}, & i=0,\ldots ,M \end{array}$$

with *p* being the probability of success of the Bernoulli random variables, *i.e.*, *p* = *P*_FA,SU_ or *p* = *P*_D,SU_.



**Algorithm 1** Probability mass function (PMF) of the noiseless received symbols.
 Input matrix **S** **for**
*l* = 1…2*^M^*
**do**  Compute 
$w_{l}={\text{∑}}_{k=1}^{M}S_{k,l}$ **end for** **for**
*l* = 1…2*^M^*
**do**  Compute 
$d_{l}={\text{∑}}_{z=1}^{2^{M}}\left(w_{z}=w_{l}\right)$  Compute 
$v_{l}={\text{∑}}_{k=1}^{M}h_{k}(2S_{k,l}-1)\sqrt{E_{\text{b}}}$  Compute *q_l_* = *b_w_l__*/*d_l_* **end for** The values of the weighted sum PMF are in the vector **v** = [*v*_1_, …, *v*_2_*_^M^_*]^T^. The corresponding probabilities are in the vector **q** = [*q*_1_, …, *q*_2_*_^M^_*]^T^. For convenience, sort **v** in ascending order, and sort **q** accordingly.


If *h_k_* = 1, Algorithm 1 will still return 2*^M^* amplitudes, but only *M* + 1 will be different, and the corresponding probabilities are the sum of the probabilities associated with the equal amplitudes, which specializes for the Binomial distribution.

### 3.2. Decision Thresholds

In the case of *h_k_* = 1, the values of ${\text{∑}}_{k=1}^{M}h_{k}s_{k}$ for *H*_0_ and *H*_1_ lead to disjoint sets of levels, which means that, under the ML decision rule, the FC will decide in favor of *H*_1_ if *r* is greater than or equal to a decision threshold *λ* and in favor of *H*_0_, otherwise, with $\lambda =(2K-M-1)\sqrt{E_{\text{b}}}$. In the case of fading reporting channels, the sets of levels can become superimposed, and more than one decision threshold can exist. This is clarified in what follows. Firstly, notice that applying the natural logarithm to both sides of Inequality (2) does not change the decision. Secondly, applying the analytic approximation of the max function to the log-sum-exp function (logarithm of the sum of the exponential) [21], *i.e*., ln Σ*_i_* exp(*a_i_*) ≃ max(*a_i_*), we can rewrite Inequality (2) as: decide in favor of *H*_1_ if:

(7) $$\underset{\text{s}\in D_{1}}{\min}{\left|r-\overset{M}{\underset{k=1}{\text{∑}}}h_{k}s_{k}\right|}^{2}\leq \underset{\text{s}\in D_{0}}{\min}{\left|r-\overset{M}{\underset{k=1}{\text{∑}}}h_{k}s_{k}\right|}^{2}$$

**Figure 1.**
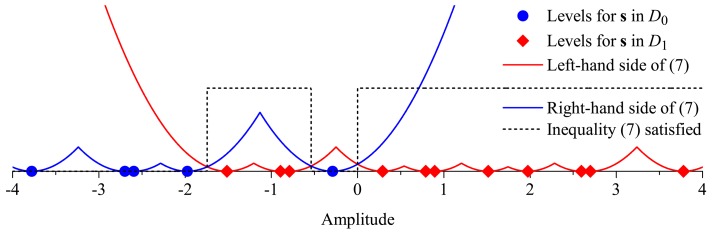
A realization of the received symbols (or levels), the likelihood functions and decision regions. Real valued channel gains are considered.

Figure 1 depicts the values of ${\text{∑}}_{k=1}^{M}h_{k}s_{k}$ corresponding to **s** ∈ *D*_1_ (diamonds) and **s** ∈ *D*_0_ (dots), for *E*_b_ = 1, *M* = 4, *K* = 2 and **h** = [*h*_1_, …,*h_M_*]^T^ = [0.592, 0.902, 1.744, 0.540]^T^. Also plotted in this figure are the values of the left-hand side and right-hand side of Inequality (7), with emphasis on the situations in which the Inequality (7) is satisfied (high levels of the dashed line). From this figure, one can observe that the decision thresholds are in the midpoints between neighbor levels pertaining to different sets. Notice that, to some extent, this model can be associated with a 2*^M^*-ary pulse amplitude modulation (PAM) constellation [20] from which a set of symbols (or levels) is associated with the hypothesis *H*_0_ and the other set is associated with the hypothesis *H*_1_. The numbers of elements in the sets of decisions *D*_1_ and *D*_0_ are, respectively:

(8) $$\begin{array}{cc} {\text{L}}_{1}=\overset{M}{\underset{i=K}{\text{∑}}}\left(\begin{array}{c} M\\ i \end{array}\right), & {\text{L}}_{0}=2^{M}-{\text{L}}_{1} \end{array}$$

The number *N* of thresholds is always odd; a sketch of the proof goes as follows, taking Figure 1 as an exemplifying reference: since *K* ≥ 1, the leftmost level corresponds to the all-zero state of {*m_k_*} and will always belong to *D*_0_; similarly, the rightmost level corresponds to the all-one state of {*m_k_*} and will always belong to *D*_1_. For each level or group of levels belonging to *D*_1_ and located in-between levels belonging to *D*_0_, there will be a rising-edge transition and a falling-edge transition in the dashed line, identifying an even number of thresholds. This even number plus the threshold related to the last rising-edge transition occurring after the rightmost level pertaining to *D*_0_ will finally result in an odd value for *N*.

A simple and fast procedure for determining the thresholds is by applying Algorithm 2, where the matrix **Z** of order 2*^M^* × 2 is formed by two arrays: the first array has its first column formed by the levels pertaining to *D*_0_ and its second column formed by zeros; the second array has its first column formed by the levels pertaining to *D*_1_ and its second column formed by ones. These two arrays are stacked, with the first array above the second, and the elements in the first column of the resultant array are sorted in ascending order; the elements in the second column are sorted accordingly. The resultant matrix is **Z**.



**Algorithm 2** Finding the decision thresholds.
 Input matrix **Z** Set the variables *u* = 0 and *λ* = 0 **for**
*i* = 1…2*^M^* − 1 **do**  **if**
*Z_i_*_,2_ ≠ *Z_i_*_+1,2_
**then**   Compute 
$\lambda_{u}=\frac{Z_{i,1}+Z_{i+1,1}}{2}$   Do *u* ← *u* + 1  **end if** **end for** The thresholds are in the vector *λ* = [*λ*_1_, …, *λ_N_*]^T^.


### 3.3. Probabilities of Detection and False Alarm

The theoretical probabilities of detection and false alarm can be computed for a given channel realization as the areas of the Gaussian tails inside each decision region, weighted by the probabilities of the corresponding levels. With the help of Figure 1, it can be seen that these probabilities can be computed from:

(9) $$P=\overset{\frac{N-1}{2}}{\underset{i=1}{\text{∑}}}\left[\overset{2^{M}}{\underset{l=1}{\text{∑}}}Q\left(\frac{\lambda_{2i-1}-v_{l}}{\sigma }\right)q_{l}-\overset{2^{M}}{\underset{l=1}{\text{∑}}}Q\left(\frac{\lambda_{2i}-v_{l}}{\sigma }\right)q_{l}\right]+\overset{2^{M}}{\underset{l=1}{\text{∑}}}Q\left(\frac{\lambda_{N}-v_{l}}{\sigma }\right)q_{l}$$

where {*v_l_*} and {*q_l_*} are computed via Algorithm 1, with {*q_l_*} being dependent on which probability we are interested in, as defined by Equation (6). The thresholds {*λ*} are computed via Algorithm 2. If *E*_b_/*N*_0_ is in dB, the noise variance is given by *σ*^2^ = *E*_b_/(2 × 10*^E^*^_b_/10^*^N^*^_0_^).

If a time-varying, real-valued, reporting fading channel is considered, we just have to average the results obtained from Equation (9) over a number of channel realizations enough for the desired accuracy of the estimated probabilities.

This is in order to state that Equation (9) applies unchanged to the AWGN reporting channel, for which the channel gains are unitary. In this case, the results obtained from Equation (9) are equal to those obtained from Equations (3) and (4) and are in agreement with the corresponding results in [19].

## 4. Numerical Results for Real-Valued Channel Gains

All of the results shown hereafter regarding the system performance consider the probability of the detection and the probability of a false alarm as a function of the average SNR per bit (*E*_b_/*N*_0_) in the links from the SUs to the FC. The main objective is to access the performance of the fusion strategy from the perspective of its ability to achieve target probabilities.

As in [19], for performance comparisons, we consider here the simultaneous transmission reporting scheme and the conventional reporting scheme that sends the local decisions via orthogonal channels. For this conventional scheme, if $P_{\text{D},\text{SU}}^{\prime }$ and $P_{\text{FA},\text{SU}}^{\prime }$ respectively, denote the probability of detection and the probability of false alarm, taking into account the transmission errors for the local decisions, these probabilities are given by:

(10) $$P_{\text{D},\text{SU}}^{\prime }=P_{\text{D},\text{SU}}(1-P_{\text{e}})+P_{\text{e}}(1-P_{\text{D},\text{SU}})$$

(11) $$P_{\text{FA},\text{SU}}^{\prime }=P_{\text{FA},\text{SU}}(1-P_{\text{e}})+P_{\text{e}}(1-P_{\text{FA},\text{SU}})$$

where *P*_e_ is the modulation-dependent and channel-dependent bit error probability. For BPSK modulation with coherent detection over the AWGN reporting channel and over the slow flat Rayleigh fading reporting channel with unitary second moment gains, this probability is respectively given by: [20]

(12) $$P_{\text{e}}=Q\left(\sqrt{\frac{2E_{\text{b}}}{N_{0}}}\right)$$

(13) $$P_{\text{e}}=\frac{1}{2}\left(1-\sqrt{\frac{E_{\text{b}}/N_{0}}{1+E_{\text{b}}/N_{0}}}\right)$$

The probabilities of detection and false alarm at the FC for the conventional scheme are, then,

(14) $$P_{\text{D},\text{FC}}={\text{∑}}_{l=K}^{M}\left(\begin{array}{c} M\\ l \end{array}\right){P_{\text{D},\text{SU}}^{\prime }}^{l}{\left(1-P_{\text{D},\text{SU}}^{\prime }\right)}^{M-l}$$

(15) $$P_{\text{FA},\text{FC}}={\text{∑}}_{l=K}^{M}\left(\begin{array}{c} M\\ l \end{array}\right){P_{\text{FA},\text{SU}}^{\prime }}^{l}{\left(1-P_{\text{FA},\text{SU}}^{\prime }\right)}^{M-l}$$

For convenience, hereafter, we identify the conventional scheme as the reference and the scheme proposed in [19] as the new scheme. For both the probabilities of detection and false alarm, the secondary users were configured to achieve the target performances *P*_D,FC_ = 0.9 and *P*_FA,FC_ = 0.1 in the error-free scenario, *i.e.*, for *E_b_*/*N*_0_ → ∞ in Equations (3), (4) and (9) and for *P*_e_ = 0 in Equations (10) and (11). All simulation results were obtained from 50,000 Monte Carlo events, each event corresponding to sending the sensing information to the FC through the reporting channel and then deciding upon the sensed channel occupation. In each Monte Carlo event, besides a new realization of the channel gains (if a fading channel is adopted) and noise, new pairs of states of the secondary user decisions are generated according to a binomial distribution with parameters *M* and *p* = *P*_D,SU_ and *p* = *P*_FA,SU_. This pair of states generates a pair of local decision vectors s, and each pair of decision vectors generates a pair of received signal values according to Equation (1). These values are used separately for computing the false alarm and the detection rates, which are the estimates of the corresponding probabilities.

The theoretical results for the AWGN reporting channel were directly computed from Equation (9). When a fading channel is considered, the theoretical results were obtained from averages of 10,000 results computed from Equation (9). These results are denoted as new, theory in the graphs.

In all results, the system parameters were chosen as *M* = 3 and *M* = 5 secondary users, for *K* = 1, *K* = ⌈*M*/2⌉ and *K* = *M* in the *K*-out-of-*M* rule. These values of *K* were chosen to configure the well-known decision fusion rules OR, majority-voting and AND, respectively.

### 4.1. Results for AWGN Reporting Channels

**Figure 2.**
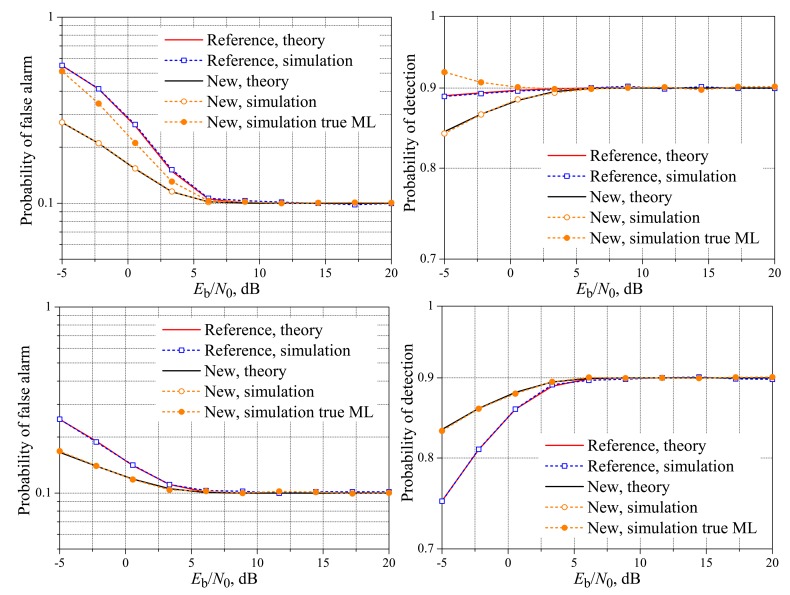

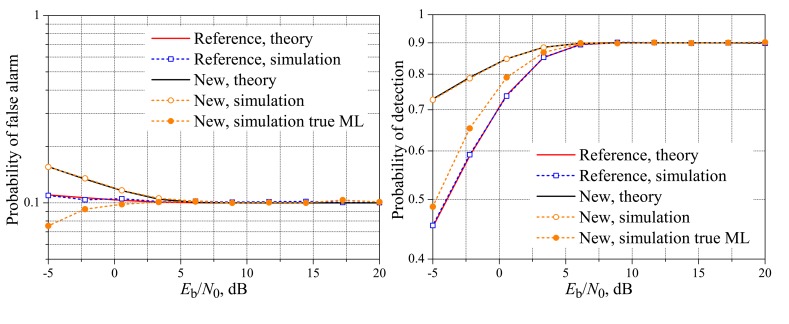
Spectrum sensing performance for the AWGN reporting channel. *M* = 3 and *K* = 1 (**top**), *K* = 2 (**middle**) and *K* = 3 (**bottom**).

**Figure 3.**
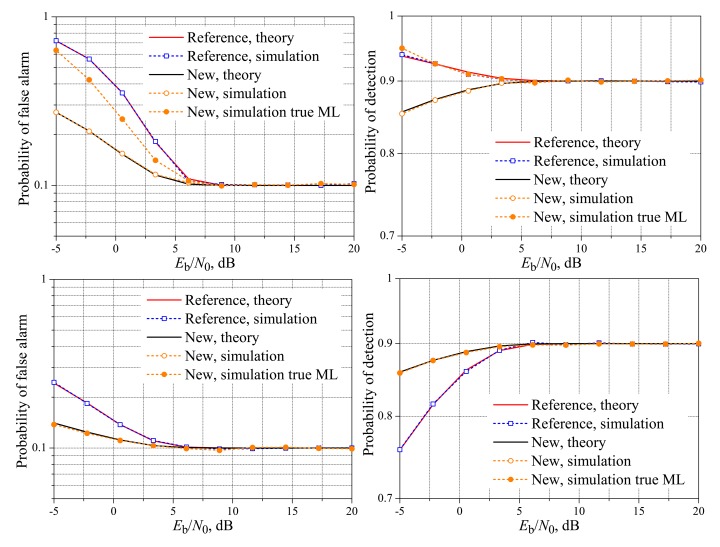

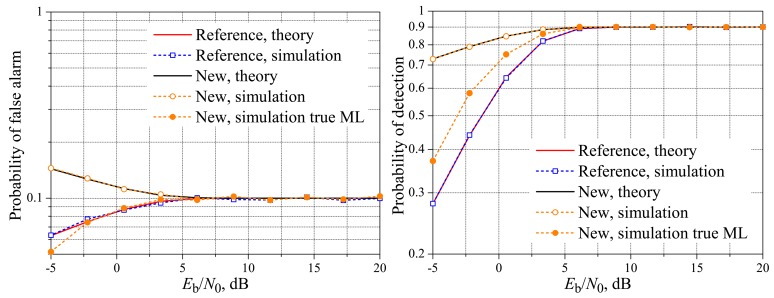
Spectrum sensing performance for the AWGN reporting channel. *M* = 5 and *K* = 1 (**top**); *K* = 3 (**middle**); and *K* = 5 (**bottom**).

Figures 2 and 3 show the theoretical and simulated performances of the spectrum sensing using the reference and the new fusion schemes over the AWGN reporting channel. It can be seen that the reference and the new schemes can closely achieve the target performances in terms of the probabilities of false alarm and detection at *E*_b_/*N*_0_ ≈ 6 dB, without regard to *K*, a conclusion also reported in [19]. However, the results in [19] are plotted only for values of *E*_b_/*N*_0_ above 6 dB; here, we extend the range of analysis by also taking into account the important region of lower signal-to-noise ratios.

In Figures 2 and 3 and the other ones shown hereafter, the simulation results for the new scheme were plotted considering that the ML decision rules are the true one given in Inequality (2) and the approximate one given in Inequality (7). In the present context, the approximation of the max function to the log-sum-exp function is tight only for high values of *E*_b_/*N*_0_. Since this approximation was considered in [19] in the derivation of Equations (3) and (4) for the AWGN reporting channel, these expressions can be inaccurate in the low *E*_b_/*N*_0_ regime, depending on the values of *M* and *K*. This dependence can be explained by noticing that when the right-hand side of Inequality (2) leads to a function of *r* with the same shape and size of the left-hand size, the decision threshold will be in the midpoint between the sets *D*_0_ and *D*_1_, no matter the noise variance. This occurs, for instance, with *M* = 3 and *K* = 2, a case in which *D*_0_ and *D*_1_ have the same number of elements. If *M* = 3 and *K* = 1, for example, the numbers of elements in *D*_0_ and *D*_1_ will be different, sometimes leading to unequally-sized functions corresponding to the left-hand side and right-hand side of Inequality (2). In this situation, different values of *σ*^2^ will bring unbalanced modifications in these functions, shifting the decision thresholds away from the values obtained using Inequality (7); the larger the value of *σ*^2^, the larger the shifts.

From the results in Figures 2 and 3, it can be seen that the Equation (9) is accurate for computing the theoretical performance of the spectrum sensing for the AWGN channel for any value of *E*_b_/*N*_0_, the same accuracy provided by the Equations (3) and (4) derived in [19]. The accuracy of Equation (9) will be also verified in the next subsection, where the results for real-valued Rayleigh fading reporting channels are considered.

It is important to observe that, if the decision thresholds are computed from the crossing points between the likelihood functions corresponding to the left-hand side and the right-hand side of the true ML rule Inequality (2) and used in Equation (9), the simulation and theoretical results agree. Such results are not provided in this paper, since it is computationally costly to obtain such crossing points, which renders the approximate ML decision to be the preferred one in practice. Nevertheless, for the sake of completeness, we will also provide the simulation results of the new scheme with the true ML decision rule.

Back to the analysis of the low *E*_b_/*N*_0_ region in Figures 2 and 3, we see that the new system with the true ML decision achieves a lower probability of false alarm and a higher probability of detection than the reference system when both cannot meet the target performances. If the approximate ML decision is applied instead, its superiority regarding the probability of false alarm does not occur for *M* = *K*, and its superiority regarding the probability of detection does not occur for *M* = 1, again comparing with the reference system.

In what concerns the influence of *M* on the new scheme with the approximate ML decision, for *K* = 3 and *K* = 1, there is a negligible performance difference from *M* = 3 to *M* = 5, with a small advantage of *M* = 5, both in terms of the probability of false alarm and the probability of detection. No pattern was identified when comparing the performances of the new scheme using the true ML decision with different values of *M*. The performance gaps between the new and the reference scheme have also increased from *M* = 3 to *M* = 5, for both the approximate and the true ML decisions. These performance gaps increase as *E*_b_/*N*_0_ decreases, with an advantage (lower false alarm and higher detection rates) for the new scheme with the true ML decision.

### 4.2. Results for Real-Valued Rayleigh Reporting Channels

**Figure 4.**
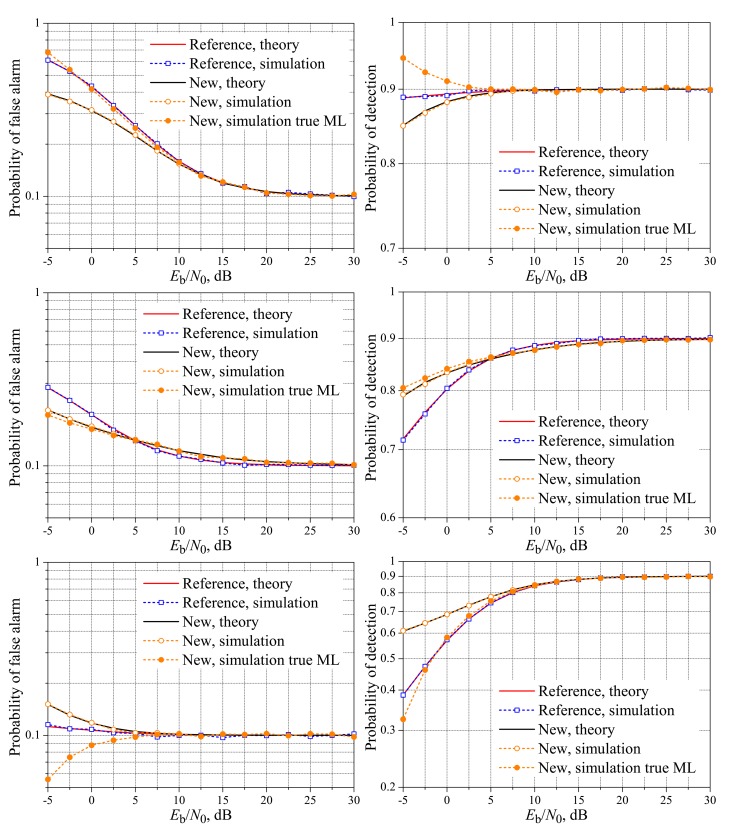
Spectrum sensing performance for the real-valued Rayleigh reporting channel. *M* = 3 and *K* = 1 (**top**); *K* = 2 (**middle**); and *K* = 3 (**bottom**).

**Figure 5.**
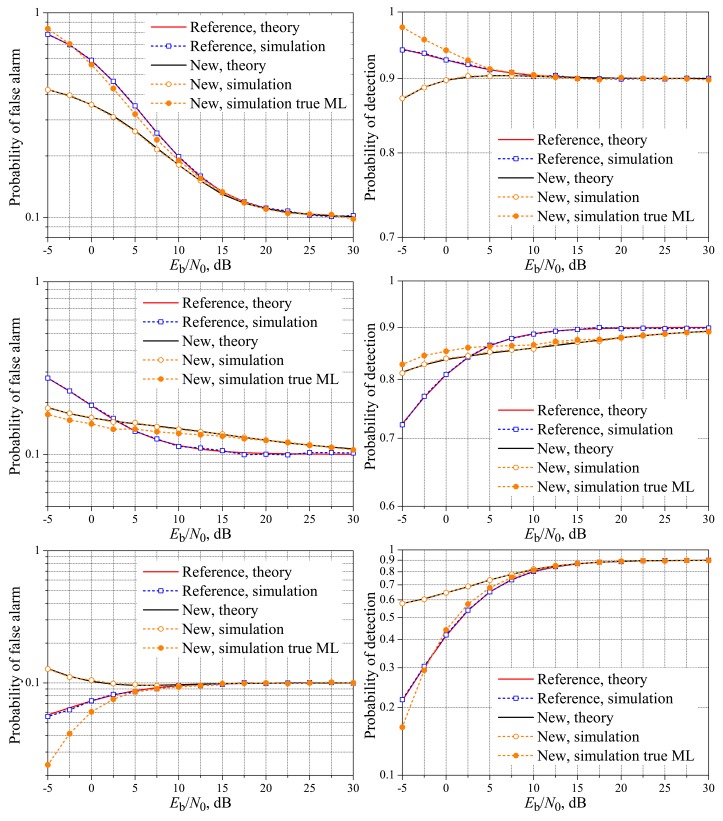
Spectrum sensing performance for the real-valued Rayleigh reporting channel. *M* = 5 and *K* = 1 (**top**); *K* = 3 (**middle**); and *K* = 5 (**bottom**).

Figures 4 and 5 show the theoretical and simulated performances of the reference and the new fusion schemes over real-valued Rayleigh fading reporting channels, again considering *M* = 3, *M* = 5 and variable *K*. Now, it can be seen that the reference and the new schemes can achieve the target performances for quite different values of *E*_b_/*N*_0_. For instance, the target probability of detection is reached at *E*_b_/*N*_0_ ≈ 6 dB for both *M* = 3 and *M* = 5, with *K* = 1, and the target false alarm probability is reached also at *E*_b_/*N*_0_ ≈ 6 dB, but for *K* = *M*. The worst situation occurs when *K* = 2 with *M* = 3 and *K* = 3 with *M* = 5, in which the new scheme reaches the target measures for significantly higher values of *E*_b_/*N*_0_: in the specific case of *M* = 5 and *K* = 3, the target performances are achieved by the new system approximately 15 dB above the reference scheme, although the performance gaps are not too high in lower values of *E*_b_/*N*_0_, up to around 5 dB, where these gaps start to increase similarly to what has been observed for the AWGN reporting channels. Nevertheless, the value of *E*_b_/*N*_0_ above which both techniques achieve the target performances for any system parameter is by far greater than in the case of the AWGN channel, which is an expected result, since the error probability over the fading channel is larger than the error probability over the AWGN channels for the same average *E*_b_/*N*_0_. The conclusions regarding the low *E*_b_/*N*_0_ regime for the AWGN channels also apply to real-valued Rayleigh fading channels.

## 5. Receiver Structure in Complex-Valued Reporting Channel Gains

**Figure 6.**
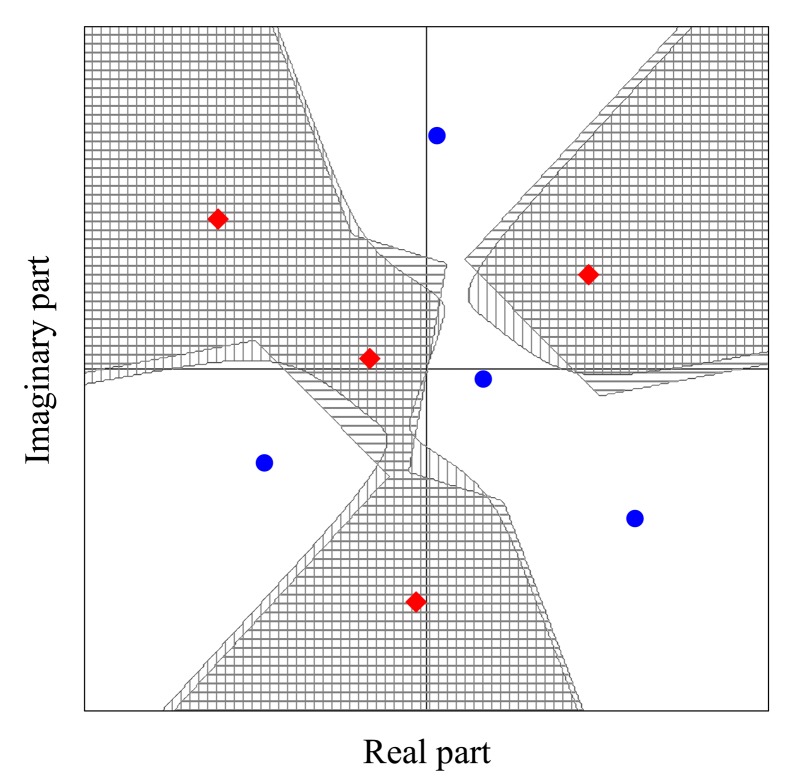
Received symbol constellation and decision regions for a given channel realization. Shaded areas with horizontal lines correspond to *H*_1_ and the approximate ML rule. Shaded areas with vertical lines correspond to *H*_1_ and the true ML rule.

In this section, we consider the most realistic situation in which the reporting channel gains are complex-valued and possibly time-varying. The picture changes significantly when compared to the channels with real-valued gains, as can be seen from Figure 6, where the noiseless received symbols (dots for *D*_0_ and diamonds for *D*_1_) are plotted for single realizations of complex Gaussian reporting channels, which correspond to Rayleigh fading channels. In this plot, we have adopted *M* = 3, *K* = 2 and **h** = [*h*_1_, …, *h_M_*]^T^ = [−0.9598 − *j*0.3668, −0.2029 + *j*1.0683, 0.6646 − *j*0.6109]^T^. Also plotted in Figure 6 are the decision regions defined from the true ML rule Inequality (2) (shaded with vertical lines for *H*_1_) and the approximate ML rule Inequality (7) (shaded with horizontal lines for *H*_1_). The true ML regions were plotted for *E*_b_/*N*_0_ ≈ 0 dB, a value for which the difference in using Inequalities (2) or (7) starts to becomes evident, for the considered system parameters. Due to the irregularity and randomness of the shape of the decision regions, the derivation of the probabilities of false alarm and detection is much more challenging than in the case of real-valued gains, even if these gains are fixed but drawn from a given distribution. If the approximate ML rule is applied, the decision boundaries can be seen as the boundaries of adjacent Voronoi regions defined by symbols from different sets. In this case, the theoretical analysis is facilitated, but it is still intricate.

It can happen that two closely-nearby signal points belong to different hypotheses, no matter how large the SNR is. Under such a circumstance, the instantaneous performance is expected to be severely degraded, as also happens, for instance, in a deep fading condition. This is a normal and expected situation, since, on average, the performance can be the desired one.

We now address the receiver structure, which makes use of the approximate ML decision rule Inequality (7) in its vector form. This rule is preferred over the true ML rule, since it is simpler from the implementation viewpoint. Arranging the symbols from the sets of decisions *D*_0_ and *D*_1_ in matrices of orders 2 × 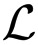
_0_ and 2 × 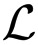
_1_, respectively, we have:

(16) L0=[Re(v0T)Im(v0T)],L1=[Re(v1T)Im(v1T)]

where the elements of **v**_0_ ∈ ℂ^
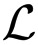
_0_×1^ are given by **h**^T^**s** for all **s** ∈ *D*_0_ and the elements of **v**_1_ ∈ ℂ^
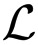
_1_ ×1^ are given by **h**^T^**s** for all **s** ∈ *D*_1_. Let ${\text{e}}_{0}=\text{diag}({\text{L}}_{0}^{\text{T}}{\text{L}}_{0})$ and ${\text{e}}_{1}=\text{diag}({\text{L}}_{1}^{\text{T}}{\text{L}}_{1})$. Additionally, let the received sample r in Equation (1) be written as **r** = [*r*_1_, *r*_2_]^T^ = [Re(*r*), Im(*r*)]^T^. Then, it can be shown that, under the simplified ML decision rule Inequality (7), the decision in favor of *H*_1_ will be made if the following inequality holds:

(17) $$\max\left[{\text{L}}_{1}^{\text{T}}\text{r}-\frac{1}{2}{\text{e}}_{1}\right]\geq \max\left[{\text{L}}_{0}^{\text{T}}\text{r}-\frac{1}{2}{\text{e}}_{0}\right]$$

The approximation of the log-sum-exp function adopted to derive Inequalities (7) and (17) is tight only if the components of the argument of the max function are not very close to each other, which corresponds to high *E*_b_/*N*_0_ values. However, when *E*_b_/*N*_0_ decreases, these components tend to become approximate in value, and in this case, there is a closer bound, which is tighter and is given by [21]:

(18) $$\ln\overset{\text{L}}{\underset{i=1}{\text{∑}}}\exp(a_{i})≃\max(a_{i})+\ln(\text{L})$$

Applying this new bound to Inequality (17), we have an improved approximate ML decision rule, which is expected to be more accurate for low signal-to-noise ratios. In this improved rule, the decision in favor of *H*_1_ will be made if the following inequality holds:

(19) $$\max\left[{\text{L}}_{1}^{\text{T}}\text{r}-\frac{{\text{e}}_{1}}{2}\right]+\frac{\ln({\text{L}}_{1})}{2}\geq \max\left[{\text{L}}_{0}^{\text{T}}\text{r}-\frac{{\text{e}}_{0}}{2}\right]+\frac{\ln({\text{L}}_{0})}{2}$$

**Figure 7.**
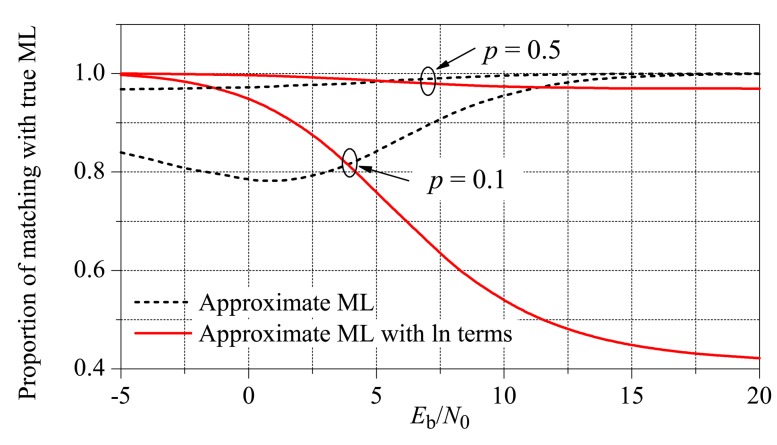
Proportion of matching between the true ML decisions and the approximate ML decisions with (solid) and without (dashed) correction for the low *E*_b_/*N*_0_ regime.

In the above inequality, the factors 1/2 in the ln terms were inserted to compensate for a division by two, which has been made in the derivation of Inequality (17). The terms added do not produce any change in the decision if *M* and *K* are chosen such that 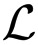
_0_ = 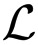
_1_. To illustrate the effect of these terms, in Figure 7 are plotted the proportions of matching between the true ML decisions made by Inequality (2) and the approximate ML decisions without (dashed, from Inequality (17)) and with (solid, from Inequality (19)) the correction terms. The system parameters were *M* = 5, *K* = 1, and the probabilities of declaring the hypothesis *H*_1_ was *p* = 0.1 and *p* = 0.5. From this figure, we can see that the receiver can be adapted to the value of *E*_b_/*N*_0_ by using or not using the correction terms in the approximate ML decision rule. If adaptation is used, the correction terms must be added if the instantaneous received SNR per bit ‖**h**‖^2^*E*_b_/*N*_0_ falls below a reference value. This value can be empirically determined for a given set of system parameters, using curves similar to those in Figure 7. As a rule-of-thumb, it can be determined from the average of the crossing points when the probabilities of declaring the hypothesis *H*_1_ are zero and one. Applying this rule for *M* = 3 and *M* = 5, the reference values were found to be around 2.6 dB and 6 dB, respectively, both for *K* = 1 and *K* = *M*.

**Figure 8.**
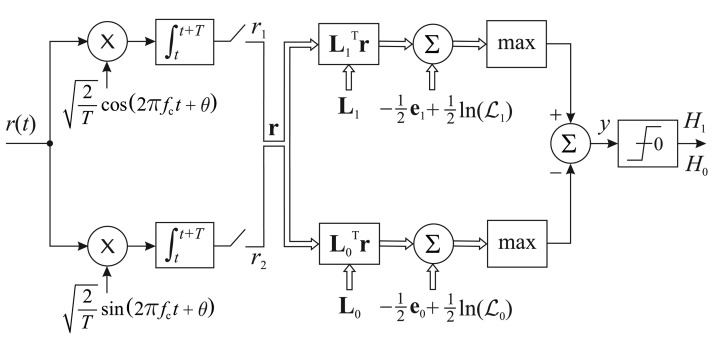
Suboptimum receiver with improved performance in low SNR.

Putting it all together, the receiver at the fusion center can be constructed according to Figure 8. The quadrature carriers do not need to be in phase coherence with the carriers used for transmitting the SUs' decisions. Indeed, it is hard to establish phase coherence with any of the SUs' carriers, since the signals from the SUs are incoherently added at the FC antenna. Nevertheless, the initial phase (say, zero) of the quadrature carriers at the beginning of the interval *T* corresponding to the reception of the SUs' signals must be equal to the initial phase of the carriers used by the SUs at the beginning of their transmissions. Since the SUs' transmitters and the FC receiver are synchronized with respect to the start and end of the interval *T*, this is easy to implement in practice. The restriction of equal initial phases is necessary to establish the correct reference for the expected received symbol positions in the constellation, which is determined by the knowledge of the reporting channel gains and of the sets *D*_0_ and *D*_1_.

## 6. Numerical Results for Complex-Valued Channel Gains

In order to obtain the results shown in this section, we have adopted the same simulation setup and system parameters adopted for the case of real-valued channel gains. First, we compare the performances of the reference system and the technique proposed in [19] with the true and the approximate ML decision rules, similarly to what we have done based on Figures 4 and 5. Then, we give a closer look at the low *E*_b_/*N*_0_ region and check the performances of the true ML decision rule against the approximate ML rule and the improved ML rule provided by the receiver in Figure 8.

### 6.1. Performances of the Reference and the New System with the True and Approximate ML Decisions

**Figure 9.**
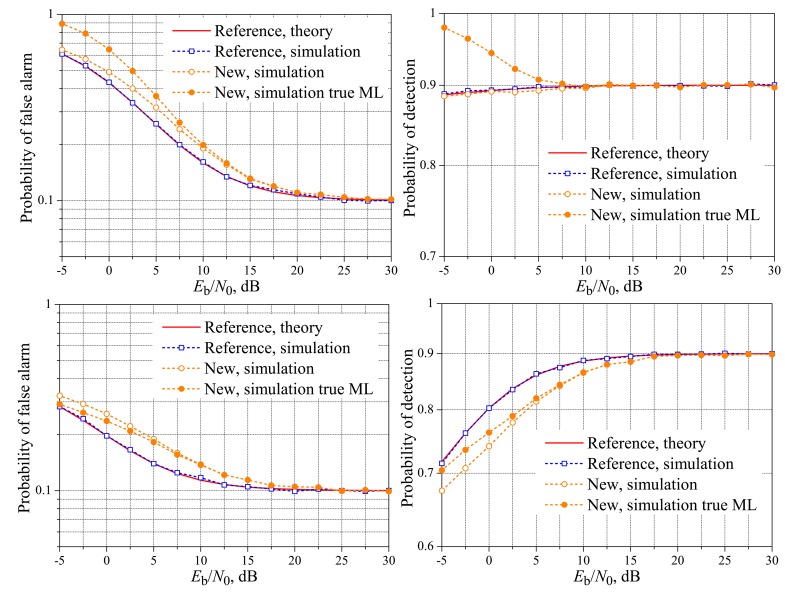

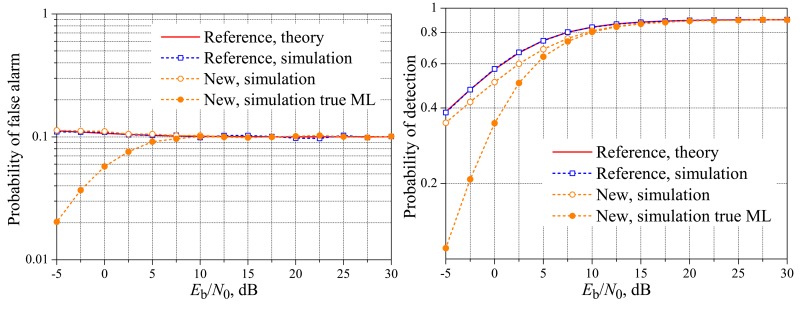
Spectrum sensing performance for the complex-valued Rayleigh reporting channel. *M* = 3 and *K* = 1 (**top**); *K* = 2 (**middle**); and *K* = 3 (**bottom**).

**Figure 10.**
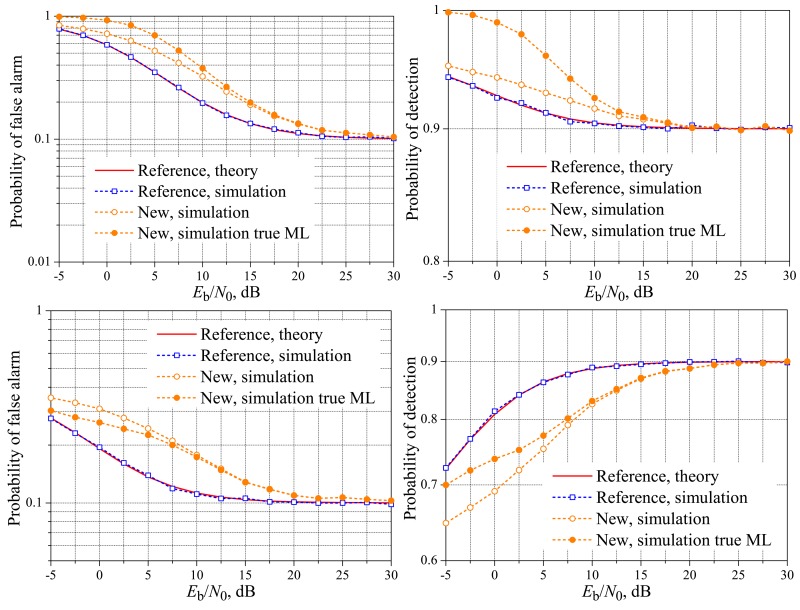

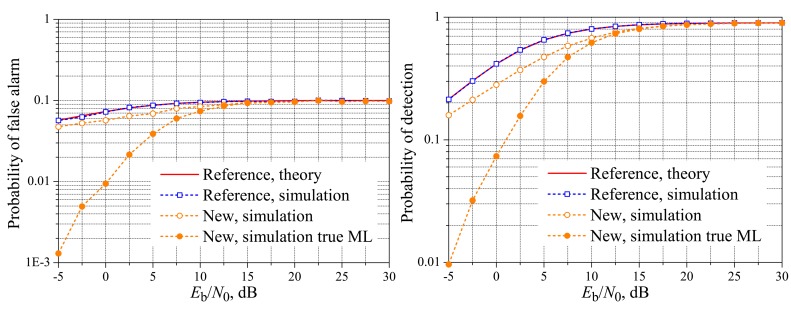
Spectrum sensing performance for the complex-valued Rayleigh reporting channel. *M* = 5 and *K* = 1 (**top**); *K* = 3 (**middle**); and *K* = 5 (**bottom**).

Figures 9 and 10 show the theoretical and simulated performances of the reference system and the simulated performances of the new fusion scheme over complex Gaussian (Rayleigh fading) reporting channels, for *M* = 3, *M* = 5 and variable *K*. As in the case of real-valued fading channels, it can be seen that the reference and the new schemes can achieve the target performances for different values of *E*_b_/*N*_0_, but the difference is not as high as in the case of real-valued channels. The worst situation again is observed for *M* = 5 and *K* = 3, for which the target performance of the new system is achieved around 10 dB above the reference system. All performances together reach the targets approximately at the same value of *E*_b_/*N*_0_, as happened in the case of real-valued gains, with the exception of the probability of detection for *M* = 5 and *K* = 1, a case in which the target is achieved around 20 dB, against 7 dB for the real-valued channel gains. The gaps between the performances of the true ML rule and the approximate one are apparent in low SNR values, with increasing gaps in lower SNRs. Especially for *M* = 5 and *K* = 5, the gaps are significantly larger in the lower SNRs than in the case of real-valued gains. It can also be seen from Figures 9 and 10, especially from Figure 10, that the situations corresponding to the majority-voting rule lead to considerable superiority in the performance of the reference system, i.e., larger *P*_D,SU_ and smaller *P*_FA,SU_. Finally, it is important to mention that the performances of the reference system and the new system with the true ML rule are in agreement with the corresponding ones in [19], recalling that the performances for the new system with the approximate decision rule were not considered in [19], except for the high SNR values where the true and approximate rules perform comparably. The range of *E*_b_/*N*_0_ considered in [19] is from 6 to 20 dB.

### 6.2. Performance of the Adaptive Receiver

**Figure 11.**
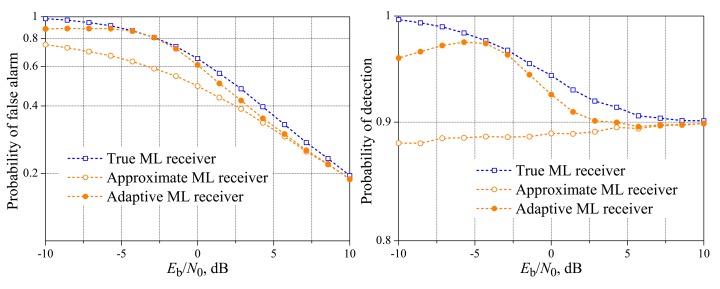

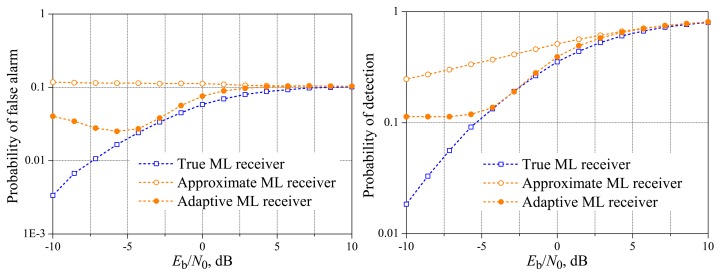
Performances of the improved ML (adaptive), true ML and approximate ML receiver for: *M* = 3 and *K* = 1 (**top**); and *M* = 3 and *K* = 3 (**bottom**).

**Figure 12.**
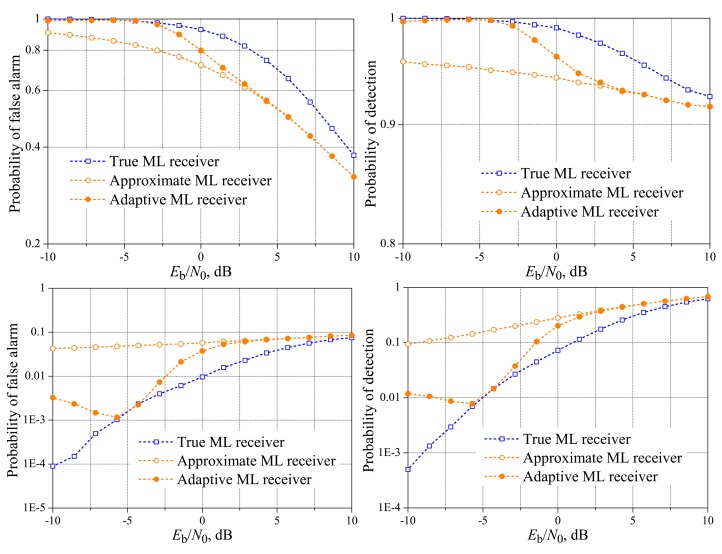
Performances of the improved ML (adaptive), true ML and approximate ML receiver for: *M* = 5 and *K* = 1 (**top**); and *M* = 5 and *K* = 5 (**bottom**).

Figures 11 and 12 show the performances of the true ML decision rule against the approximate ML rule and the improved ML rule provided by the adaptive receiver shown in Figure 8, with emphasis on the low SNR regime. In the case of *M* = 3, the instantaneous reference value of *E*_b_/*N*_0_, below which the logarithmic terms are added to the approximate ML rule according to Inequality (19), was set to 2.6 dB. For *M* = 5, this value was set to 6 dB. These values were found according to the procedure described in Section 5. From these figures, we readily see that the adaptive receiver is effective at approximating its performance to the one achieved by the true ML decision rule of [19], in low values of *E*_b_/*N*_0_, with the potential for improving the overall performance of the spectrum sensing. This improvement will correspond to a better *P*_FA,FC_ *versus* a *P*_D,FC_ tradeoff in situations where the target performance cannot be achieved.

The cases of *M* = 3 and *K* = 2 and *M* = 5 and *K* = 3 were omitted in this subsection, because 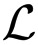
_0_ = 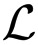
_1_, and the adaptation of the receiver does not bring any performance change.

## 7. Conclusions and Final Remarks

This paper extended the results of [19], where a novel technique for sending the spectrum sensing information of secondary users to the fusion center in a cooperative spectrum sensing has been proposed. In this technique, the secondary users simultaneously transmit their local decisions in the same frequency, saving time and frequency resources.

In this paper, we have derived the probabilities of detection and false alarm related to the technique of [19], considering real-valued fading reporting channels and the maximum likelihood decision. Simulation and analytical results were shown for both the AWGN and the real-valued Rayleigh fading channels, demonstrating the accuracy of the derived expression. Our results have unveiled that, from a practical standpoint, the new technique and the conventional or reference one, which uses orthogonal channels to transmit the local decisions, can reach performances that are not far from the target in approximately the same signal-to-noise ratios. This is an attractive aspect of the fusion scheme proposed in [19], since it is capable of using less resources for reporting the sensing information gathered by the secondary users. If some further improvement in performance is needed, mainly in the low signal-to-noise ratio regime, the transmit powers of the secondary users can be increased in a traded fashion regarding the time or frequency resources available. On the other hand, the energy efficiency of the system can be improved if the performance can be relaxed, compared with the reference system, by reducing the average transmitted powers of the secondary user's terminals.

We have also proposed an adaptive receiver that is capable of approximating the performance of the true maximum-likelihood decision rule in the low signal-to-noise ratio regime, with a negligible increase in complexity.

New information regarding the technique proposed in [19] was also provided throughout this paper, allowing for a better and complete understanding about it.

Due to the irregularity of the shape of the decision regions in the case of complex-valued channel gains and to their randomness due to the time variability of fading channels, the derivation of the probabilities of detection and false alarm becomes a challenging task, representing an interesting opportunity for future work.
